# Molecular mechanism of apoptosis induction in skin cancer cells by the centipedegrass extract

**DOI:** 10.1186/1472-6882-13-350

**Published:** 2013-12-11

**Authors:** Srilatha Badaboina, Hyoung-Woo Bai, Chul-Hong Park, Dong Min Jang, Bo Yun Choi, Byung Yeoup Chung

**Affiliations:** 1 Advanced Radiation Technology Institute (ARTI), Korea Atomic Energy Research Institute (KAERI), Jeongeup-si, Jeollabuk-do 580-185, Republic of Korea; 2 School of Biological Sciences and Biotechnology, Chonnam National University, Gwangju 500-757, Republic of Korea

**Keywords:** Centipedegrass extract, Apoptosis, B16F1, SKMEL-5, PI3K/AKT/GSK-3β, Caspase, Skin cancer

## Abstract

**Background:**

Centipedegrass extract (CGE) is mainly composed of maysin and its derivatives, which are recognized internationally as natural compounds. Compared to other flavonoids, maysin has a unique structure in that mannose is bound to the flavonoid backbone. CGE exhibits some biological properties in that it can function as an anti-oxidant, anti-inflammatory, anti-adipogenic, and insecticidal. Whether CGE has other biological functions, such as anti-cancer activity, is unknown.

**Methods:**

B16F1 (mouse) and SKMEL-5 (human) cells were treated with CGE, and their subsequent survival was determined using MTT assay. We performed a cell cycle analysis using propidium iodide (PI), and detected apoptosis using double staining with annexin V-FITC/PI. In addition, we examined mitochondrial membrane potentials using flow cytometry, as well as signaling mechanisms with an immunoblotting analysis.

**Results:**

CGE inhibited skin cancer cell growth by arresting the cell cycle in the G_2_/M phase, and increased both early and late apoptotic cell populations without affecting normal cells. Furthermore, we observed mitochondrial transmembrane depolarization, increased cytochrome-c release, caspase-3 and caspase-7 activation, and increased poly ADP-ribose polymerase degradation. CGE also downregulated activation of p-AKT, p-glycogen synthase kinase-3β (GSK-3β), and p-BAD in a time-dependent manner. LY294002 inhibition of phosphoinositide 3-kinase (PI3K) significantly sensitized skin cancer cells, which led to an increase in CGE-induced apoptosis.

**Conclusions:**

CGE controlled skin cancer cell growth by inhibiting the PI3K/AKT/GSK-3β signaling pathway and activating the effector caspases. This study is the first to demonstrate anti-cancer properties for CGE, and that CGE may be an effective therapeutic agent for treating skin cancer.

## Background

Non-melanoma skin cancer has the highest incidence rate among all cancers [1]. In the US alone, more than 1 million cases are diagnosed every year, which is equivalent to the incidence of malignancies in all other organs combined [2]. The phosphoinositide 3-kinase (PI3K) pathway is frequently targeted in the germ line for somatic mutations in many human cancers. These findings, and the fact that PI3K and other kinases in the PI3K pathway are highly suitable for pharmacological studies, make this pathway one of the most attractive targets for therapeutic intervention in cancer treatment [3]. The PI3K/AKT signaling pathway participates in melanogenesis of B16 skin cancer cells [4]. However, several downstream substrates of the PI3K/AKT signaling pathway, such as glycogen synthase kinase-3β (GSK-3β), BAD, and BAX, contribute to chemotherapeutic resistance in cancer cells and regulated apoptosis.

Interest in using naturally occurring compounds for medicinal treatments is growing due to the adverse effects that are sometimes associated with non-naturally occurring medicines. Centipedegrass (*Eremochloa ophiuroides* [Munro] Hack) is a grass that is native to China and Southeast Asia, and has become one of the most popular lawn grasses in South America [5,6]. Previous analysis with liquid chromatography-mass spectrometry has identified maysin as a component of centipedegrass, in addition to maysin derivatives such as luteolin, orientin, isoorientin, rhamnosylisoorientin, derhamnoslymaysin, and luteoin-6-*C*-boivinopyranose [7]. Centipedegrass extract (CGE) also contains several C-glycosyl flavones and phenolic constituents. However, there is limited information on the biological function of CGE. For example, the methanolic extract of centipedegrass leaves exhibits pancreatic lipase inhibitory activity [7], and CGE exhibits anti-adipogenic activity and can attenuate expression of adipogenesis-related factors and lipid metabolic genes [8].

In the present study, we explored the anti-cancer activity of CGEs by applying CGE to several cancer cell lines derived from the breast, kidney, liver, prostate, and skin. Among the cells analyzed, skin cancer cells showed a particularly strong response to CGE without affecting normal cells, Detroit 551 (ATCC CCL-110) (Table 1). Therefore, we further investigated the anti-cancer properties of CGE in skin cancer cells. To better understand the mechanisms that mediate CGE actions, we analyzed various signaling pathways, particularly the PI3K/AKT/GSK-3β pathway. Given that apoptosis is an intricate process, a detailed understanding of the molecular mechanisms involved in CGE-induced apoptosis in skin cancer cells is a critical step for using CGE in cancer therapy.

**Table 1:** The anti-proliferation effects of CGE extracts on different cancer cell lines

**Cell lines**	**IC**_**50 **_**(μg/ml)**
Detroit 551 (Fibroblast cells)	> 500
MCF-7 (Breast cancer cells)	82.4 ± 3.2
HepG2 (Liver cancer cells)	74.6 ± 5.6
LNCap (Prostate cancer cells)	95.4 ± 3.1
293 T (Kidney cancer cells)	69.3 ± 2.7

Cell viability was measured by MTT assay after CGE treatment. The half-maximal inhibitory concentrations (IC_50_) were calculated using Sigma Plot 10.0 software (Systat Software Inc., San Jose, CA, USA) with the 4 parameters logistic function standard curve analysis for dose response.

## Methods

### Preparation of the CGE

Centipedegrass seeds imported from Fukukaen Nursery (Blu co. Ltd., Nagoya, Japan) were cultivated at the Korea Atomic Energy Research Institute (KAERI, Jeongeup, South Korea). The leaves of centipedegrass were harvested in October 2011 and stored at −80°C until use. The dried leaves of centipedegrass (5 kg) were ground in a Wiley mill (Weiber, India) and passed through a 420-μm sieve. The final ground sample (1 kg) was extracted three times with 80% methanol (MeOH, 100 L; Merck, Germany) for 24 h with constant shaking at ambient temperature in the dark. The extracts were filtered using No. 2 filter paper (Advantech, Japan) and concentrated *in vacuo*. The MeOH extracts were fractionated with *n*-hexane and ethyl acetate (EA), successively. The EA extracts were concentrated *in vacuo* and the dried compounds were dissolved in MeOH. The active MeOH extracts were diluted in 20% MeOH and chromatographed on a TOYOPEARL HW-40C resin (TOSOH, Japan) column using 70% MeOH (elution volume, 700 mL). The fraction was evaporated and then freeze-dried. Dried extracts were reconstituted in dimethyl sulfoxide (DMSO) for cell treatment.

### Chemicals and reagents

Thiazolyl blue tetrazolium blue (MTT), annexin V-FITC, protease inhibitor cocktail, propidium iodide (PI), and DMSO were purchased from Sigma (St. Louis, MO, USA). Antibodies for p-PI3K, p-AKT (Ser 473), p-AKT (Thr 308), AKT, p-GSK-3β (Ser 9), GSK-3β, p-BAD (Ser 136), BAD, procaspase-3, cleaved caspase-3, cytochrome-c, poly ADP-ribose polymerase (PARP), GAPDH, horseradish peroxidase (HRP)-conjugated secondary antibody, and the PI3K inhibitor LY294002 were obtained from Cell Signaling Technology (Beverly, MA, USA). The general caspase inhibitor Z-VAD-FMK was purchased from R&D Systems (Minneapolis, MN, USA). All other chemicals used in this study were obtained from Sigma.

### Cell culture

B16F1 (ATCC CRL-6323), SKMEL-5 (ATCC HTB-70), and Detroit 551 (ATCC CCL-110) lines were purchased from American Type Culture Collection (Rockville, MD, USA). Cell lines were cultured with either Dulbecco’s modified eagle’s medium (DMEM) or Eagle’s minimum essential medium (EMEM) for Detroit 551 supplemented with penicillin (100 units·mL^-1^), streptomycin (100 μg·mL^-1^), and 10% fetal bovine serum (FBS), and maintained in an incubator with a humidified atmosphere of 95% air and 5% CO_2_ at 37°C.

### Cell viability assay

Cell viability was measured using MTT. Cells were seeded in 96-well plates (1 × 10^4^ cells/well) and incubated overnight. The cells were treated with CGE at the concentrations indicated and incubated for 48 h. The cells were then incubated with 0.5 mg·mL^-1^ of MTT for 1 h at 37°C. The blue MTT formazan crystals resulting from MTT reduction were then dissolved using acidified isopropanol solubilization solution. The plates were left at room temperature for 10 min on an orbital shaker to allow for complete cell lysis. The absorbance at 570 nm was measured using a micro plate reader (Tecan, Switzerland). The half-maximal inhibitory concentrations (IC_50_) were calculated using Sigma Plot 10.0 software (Systat Software Inc., San Jose, CA, USA) with a 4-parameter logistic function standard curve analysis for dose response.

### Cell cycle analysis by flow cytometry

Skin cancer cells were seeded into 6-well plates at a density of 0.5 × 10^6^ cells/well. After 24 h, the cells were treated with 0, 25, 50, 75, and 100 μg·mL^-1^ of CGE for 48 h. The cells were collected and washed with cold 1× PBS, and then fixed in 70% cold ethanol overnight at 4°C. The fixed cells were washed and resuspended in 1× PBS containing 100 μg·mL^-1^ RNase A, incubated for 30 min at 37°C, and stained with PI (20 μg·mL^-1^) for 15–20 min at room temperature in the dark. The DNA content of the stained cells was analyzed using a FC500 flow cytometer (Beckman-Coulter, Fullerton, CA, USA). The data were analyzed using CXP analysis software version 2.2 (Beckman-Coulter, Fullerton, CA, USA).

### Apoptosis detection by annexin V/PI staining and TUNEL staining

Apoptosis can be detected by translocation of phosphatidyl serine to the cell surface using an annexin V-FITC antibody. Cells were seeded into 6-well plates (0.5 × 10^6^ cells/well), incubated overnight, treated with the indicated concentrations of CGE, and then incubated again for 48 h. To assess apoptosis, cells were washed twice with ice-cold PBS (pH 7.4), resuspended in a binding buffer (10 mM HEPES, pH 7.4, 140 mM NaCl, and 2.5 mM CaCl_2_), and incubated with annexin V-FITC for 10–15 min in the dark. PI was then added and the cells were incubated again for 15 min in the dark. Annexin V-FITC and PI fluorescence was monitored using an FC500 flow cytometer. Ten thousand events were collected per sample. Data were analyzed using CXP analysis software, and TUNEL method was applied with a commercially available DeadEnd™ Fluorometric TUNEL System (Promega Co., Madison, WI, USA) according to the manufacture’s manual to explore the CGE effect on skin cancer cells apoptosis. TUNEL positive cells were identified with a fluorescence microscope.

### Detection of mitochondrial transmembrane potential

Changes in the mitochondrial membrane potential were determined using DiOC_6_ (3). Cells were treated with DMSO or with the indicated concentrations of CGE for 48 h. Cells were then harvested, washed twice in cold 1× PBS, resuspended in 1× PBS supplemented with DiOC_6_ (3) (40 nM), incubated in the dark at 37°C in an incubator with 5% CO_2_ for 20 min, and then immediately analyzed by flow cytometry.

### Immunostaining for apoptotic proteins

CGE-treated cells were briefly washed with ice-cold 1× PBS. Cell lysis was carried out using RIPA buffer (Cell Signaling Technology, Beverly, MA, USA) with 1% (v/v) protease inhibitor, according to the manufacturer’s instructions. The protein concentrations of the supernatants were measured using bicinchoninic acid assay (Pierce, Rockford, IL, USA) with BSA as a standard. For the analysis of apoptotic proteins, equivalent amounts of protein from various samples were subjected to electrophoresis through 12% or 15% SDS-polyacrylamide gels, and subsequently transferred to polyvinyl difluoride (PVDF) membranes. The PVDF membranes were blocked with 1× Tris-buffered saline containing 0.1% Tween 20 and 5% non-fat milk at room temperature for 1 h, and then incubated overnight with the appropriate primary antibodies at 4°C on a shaker. Incubation with a HRP-conjugated secondary antibody was followed by the detection of protein expression using the ECL plus chemiluminescence kit (Amersham Biosciences, Piscataway, NJ, USA).

### Statistical analysis

The data are presented as mean ± SD for the three experiments performed in triplicate. Using Sigma Plot 10.0 software, we performed Student’s *t*-tests for the statistical analyses, and * *P* < 0.05, ** *P* < 0.01, *** *P* < 0.001 were considered statistically significant.

## Results and discussion

### CGE induced cytotoxicity through G_2_/M cell cycle arrest, and both early and late apoptosis

**Figure 1:**
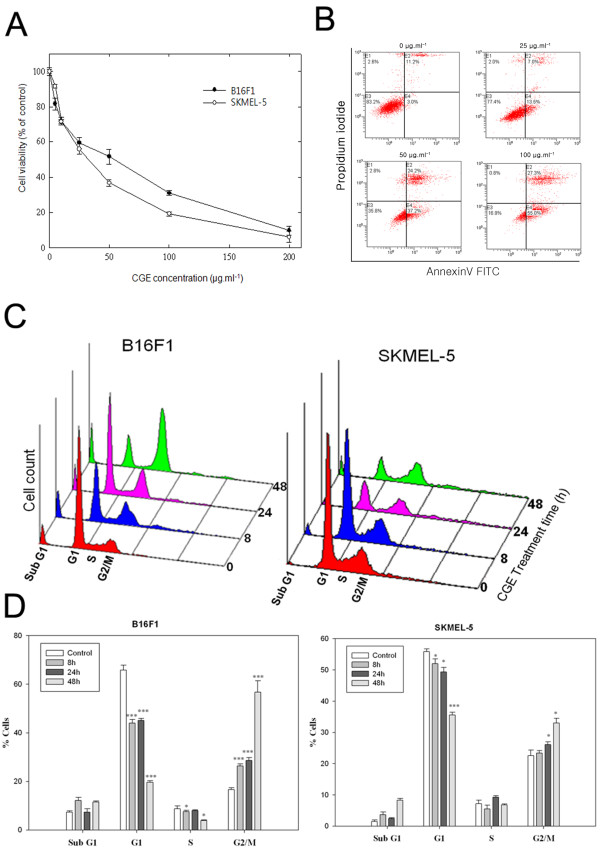
**Centipedegrass extract (CGE) induced cytotoxicity through G**_**2**_**/M cell cycle arrest and both early and late apoptosis. (A)** Cell viability of B16F1 and SKMEL-5 skin cancer cells after CGE treatment by MTT assay. **(B)** Double staining with annexin V-FITC and propidium iodide (PI) demonstrated an increase in the percentage of apoptotic cells (early and late) when skin cancer cells (SKMEL-5) were treated with the indicated doses of CGE for 48 h. **(C)** Flow cytometry analysis of cell cycle distribution in CGE-treated B16F1 and SKMEL-5 skin cancer cells. **(D)** Data are presented as mean ± SD for at least three independent experiments. * *P* < 0.05, ** *P* < 0.01, and *** *P* < 0.001 compared with the control.

CGE exhibits various biological functions [7]. The presence of the *C*-glycosidic flavonoid maysin and its derivatives suggests that CGE also may contain anti-cancer properties. We examined the anti-cancer activity of CGE in B16F1 (mouse skin cancer cell line) and SKMEL-5 (human skin cancer cell line) cells treated with varying concentrations of CGE for 48 h. We then determined the cell viability using the MTT assay. As shown in Figure 1A, CGE significantly suppressed cell proliferation in these tumor cells in a dose-dependent manner. The human skin cancer cell line (SKMEL-5) was more susceptible to CGE than the mouse skin cancer cell line (B16F1). The IC_50_ values of CGE were 19.18 and 43.41 μg·mL^-1^ for SKMEL-5, and B16F1 cells, respectively. However, the human normal fibroblast cells were not affected by CGE, suggesting that CGE might break the cell signaling pathway related with cancer cell proliferation.

To determine whether apoptosis mediated the growth inhibition observed in skin cancer cells treated with CGE, we performed an annexin V-FITC/PI double-staining experiment. A considerable increase in apoptotic cells was observed for B16F1 (78% ± 8.0%) and SKMEL-5 (69.6% ± 1.0%) cells treated with CGE (Figure 1B). Next, we performed TUNEL staining to confirm the apoptotic cells treated with CGE. CEG significantly increased TUNEL-positive nuclei in the SKMEL-5 cells, which indicates CGE induced apoptosis in skin cancer cells (Additional file 1: Figure S1).

Cell cycle checkpoints that temporarily arrest a specific cell cycle stage are important control mechanisms as they allow the cell to correct possible defects. Many anti-cancer agents halt cell cycles [9-12], which induces apoptosis in cancer cells. To examine whether CGE-induced growth inhibition is mediated by cell cycle arrest, we assessed the cell cycle distribution of skin cancer cells treated with CGE for 8, 24, or 48 h. As shown in Figure 1C–D, both skin cancer cell lines showed a significant and time-dependent G_2_/M stage arrest and a sub-G_1_ peak. A prominent population increase was observed in B16F1 cells that was time dependent. After 48 h, the cell population increased by 56.7 ± 4.80% at G_2_/M, and the sub-G_1_ peak increased from 7.3 ± 0.56% to 11.5 ± 0.49%. In the case of SKMEL-5 cells, 33.3 ± 1.50% of the cell population was arrested at the G_2_/M stage, and the sub-G_1_ increased from 1.5 ± 0.49% to 8.3 ± 0.56% (Figure 1C–D).

### CGE induced of cytochrome-c release, activated caspases, and cleaved PARP

**Figure 2:**
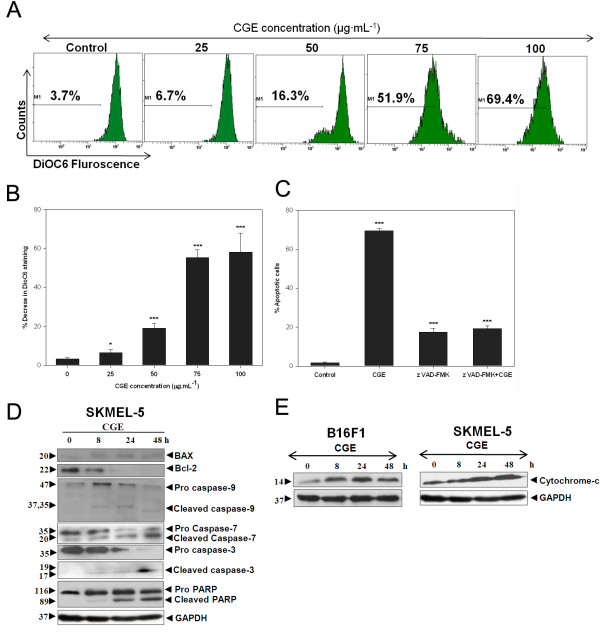
**CGE induced the release of cytochrome-c, activated caspases, and PARP. (A)** SKMEL-5 cells were treated with the indicated concentrations of CGE for 48 h, DiOC6 (3) staining was performed, and the mitochondrial membrane potential of the cells were analyzed by flow cytometry. **(B)** Data are presented as mean ± SD for at least three independent experiments. * *P* < 0.05 and *** *P* < 0.001 compared with the control. **(C)** CGE inhibition caused cytotoxicity by z-VAD FMK, as demonstrated by double staining with annexin V-FITC and PI. **(D)** Skin cancer cells were exposed to CGE (50 μg·mL^-1^) for 8, 24, and 48 h. Cell lysates were prepared and assayed for BAX, Bcl-2, caspase-9, caspase-7, caspase-3, poly-(ADP-ribose)-polymerase (PARP), and GAPDH protein levels. **(E)** B16F1 and SKMEL-5 cells were treated with 50 μg·mL^-1^ CGE for 8, 24, and 48 h. Equal amounts of protein from each sample were separated by SDS-PAGE and immunoblotted with cytochrome-c and GAPDH antibodies.

Apoptosis causes the mitochondrial membrane to breakdown and release cytochrome-c into the cytosol. Therefore, we investigated whether CGE affected the mitochondrial membrane by examining the mitochondrial membrane potential. Specifically, we used the fluorescent dye DiOC6 in CGE-treated cells to identify disruption of mitochondrial membrane potential [3]. We observed decreased staining in SKMEL-5 cells from 3.3 ± 0.69% to 58.1 ± 6.5%, which indicated CGE decreased the mitochondrial membrane potential (Figure 2A–B). These findings strongly suggest that CGE induced apoptosis in skin cancer cells that was accompanied by a breakdown in the mitochondrial membrane potential.

To determine what role caspases play in CGE-induced apoptosis, we pretreated cells with a pan-caspase inhibitor (Z-VAD-FMK) with a concentration of 50 μM. Treatment with Z-VAD-FMK resulted in considerable rescue of skin cancer cells from CGE-induced apoptosis at 48 h, as measured by annexin V-FITC/PI double staining (Figure 2C). Blocking caspase activation by Z-VAD-FMK significantly suppressed CGE-induced apoptosis.

Pro-apoptotic proteins, such as BAD and BAX, can trigger the apoptotic cascade by forming pores in the mitochondrial membrane [13,14]. These membrane pores lead to an increased cytosolic concentration of cytochrome-c, which in turn activates effector caspases. Caspase-3 activation is responsible for cleaving most apoptotic substrates such as poly-(ADP-ribose) polymerase (PARP) [15,16]. In this study, we observed decreased levels of procaspase-3, procaspase-7, and procaspase-9, but increased levels of the cleaved active forms of caspase-3, caspase-7, and caspase-9. CGE induced cleavage of PARP into 115- and 89-kDa fragments through caspase-3 activation, which suggests CGE induces apoptosis by activating caspase-3. In addition, we observed CGE upregulated BAX and downregulated Bcl-2 (Figure 2D). Apoptosis increases the permeability of the outer mitochondrial membrane and cytochrome-c release, which leads to the subsequent activation of caspase-3 [17,18]. Accordingly, we analyzed the level of cytochrome-c with immunoblotting, which revealed increased cytosolic levels of cytochrome-c in CGE-treated skin cancer cells (Figure 2E).

It was of note that Bcl-2 family of proteins has been shown to play an important role in regulating epidermal homeostasis in skin cancer cells [19]. As shown in Additional file 2: Figure S2, CGE was not affected the signaling pathway in normal fibroblast cells, indicating that cancer cells were more susceptible than normal cells to CGE.

### CGE effect of on the PI3K/AKT/GSK-3β pathway

**Figure 3:**
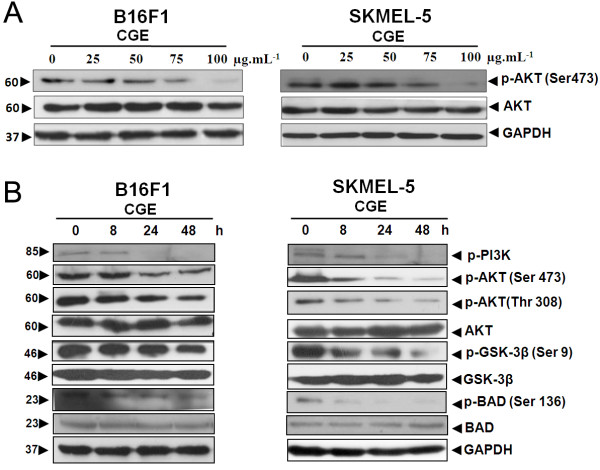
**Effect of CGE on the PI3K/AKT/ GSK-3β pathway. (A)** CGE inhibited the constitutively active PI3K/AKT/glycogen synthase kinase-3β (GSK-3β) signaling pathway in skin cancer cell lines in a dose-dependent manner. B16F1 and SKMEL-5 cells were treated with CGE at the indicated doses for 48 h. Equal amounts of protein from each sample were separated by SDS-PAGE and immunoblotted with p-AKT (Ser 473), AKT, and GAPDH. **(B)** B16F1 and SKMEL-5 cells were treated with 50 μg·mL^-1^ CGE for 8, 24, and 48 h. Proteins from each sample were separated by SDS-PAGE and immunoblotted with p-PI3K, p-AKT (Thr 308), p-AKT (Ser 473), p-GSK-3β (Ser 9), GSK-3β, p-BAD (Ser 136), BAD, and GAPDH.

The PI3K/AKT/GSK-3β signaling pathway is important for cell survival and apoptosis. AKT plays a crucial role in tumorigenesis and tumor progression by promoting cell proliferation and inhibiting apoptosis [20-22]. For example, GSK-3β is phosphorylated by AKT and helps regulate cell proliferation, cell cycle progression, and anti-apoptotic pathways [23]. Another substrate, BAD, is a pro-apoptotic member of the Bcl-2 protein family that plays an important role in apoptosis. The balance between the pro-apoptotic (e.g., BAD and BAX) and anti-apoptotic (e.g., Bcl-2 and Bcl-XL) members of the Bcl-2 family is critical for controlling mitochondria-induced apoptosis [24]. Activated caspases also can serve as biochemical markers for the apoptosis cascade reaction [25,26]. Activated AKT functions to promote cell survival by suppressing apoptosis via subsequent modulation of several target molecules that regulate apoptosis, including BAD [27-29], GSK-3β [30], and BAX [31,32]. We therefore performed and immunoblotting analysis to identify the mechanisms of CGE-induced apoptosis in skin cancer cells. As shown in Figure 3A, skin cancer cells treated with CGE (25–100 μg·mL^-1^) for 48 h exhibited a significant, dose-dependent decrease in AKT activation compared with control cells. To examine whether the effect of CGE on skin cancer cells were time dependent, we examined the phosphorylation levels of phosphorylated PI3K, AKT (Ser 473), AKT (Thr 308), GSK-3β (Ser 9), and BAD (Ser 136). The results of this analysis indicated reduced phosphorylation levels for PI3K, AKT (Ser 473), AKT (Thr 308), GSK-3β (Ser 9), and BAD (Ser 136). These decreases were accompanied by downregulation of AKT and upregulation of both GSK-3β and BAD (Figure 3B), thus indicating CGE inhibited AKT activation and activated GSK-3β and BAD.

### Combined treatment with CGE and LY294002 induced a spontaneous apoptosis rate

**Figure 4:**
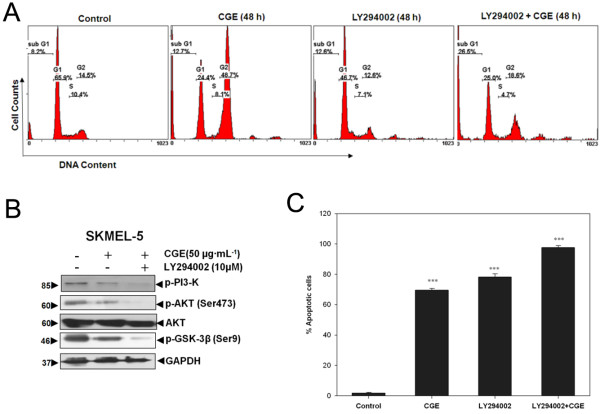
**Apoptosis induced by the combined treatment of CGE with the PI3K inhibitor LY294002. (A)** Flow cytometry analysis was used to detect the cell cycle distribution of CGE-treated skin cancer cells for 48 h in the presence or absence of LY294002 (PI3K inhibitor). **(B)** The protein levels of p-PI3K, p-AKT, p-GSK-3β, AKT, and GAPDH were examined in cells with or without CGE and LY294002 treatment. **(C)** Flow cytometry analysis of annexin V-FITC and PI double-stained cells. Data are presented as mean ± SD values for at least three independent experiments. *** *P* < 0.001 compared with the control.

CGE induced apoptosis in skin cancer cells by inhibiting the PI3K/AKT/GSK-3β pathway. Therefore, we attempted to identify PI3K contributions to CGE-induced apoptosis by treating skin cancer cells with CGE and the PI3K specific inhibitor LY294002. As shown in the example presented in Figure 4A, the sub-G_1_ peak increased from 8.2% to 12.7% with CGE treatment alone, and was further increased to 26.5% after the combined CGE and LY294002 treatment. According to the western blot analysis, we observed a significant decrease in p-PI3K, p-AKT, and p-GSK-3β expression with combined CGE and LY294002 treatment, as compared with CGE only-treated cells (Figure 4B). However, the total AKT level was unaffected, indicating that PI3K acts as an upstream factor in CGE-induced cellular pro-apoptotic signaling. To further determine the role of PI3K in CGE-induced skin cancer cell apoptosis, cells were also treated with LY294002 at a concentration of 50 μM. Treatment with CGE alone for 48 h resulted in an increase of apoptotic cells from 1.7% ± 1.0% to 69.6% ± 2.03%, whereas combined CGE and LY294002 treatment for 48 h increased the apoptotic cells from 1.7% ± 1.0% to 97.7% ± 2.0% (Figure 4C). These results indicate the PI3K inhibitor LY294002 significantly increased CGE-induced apoptosis by decreasing p-PI3K, p-AKT, and p-GSk-3β.

## Conclusions

**Figure 5:**
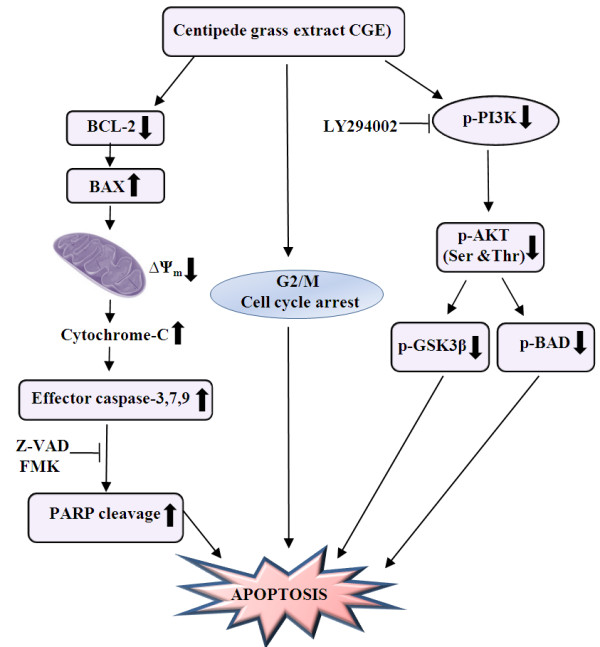
**Schematic diagram.** Schematic diagram of the apoptotic pathway induced by CGE in skin cancer cells.

In the present study, we demonstrate that CGE can affect apoptosis-associated factors in skin cancer cells. In particular, CGE-induced apoptosis was caspase dependent, and CGE downregulated p-AKT, p-GSK-3β, and p-BAD activation in a time-dependent manner without affecting normal cells. LY294002 inhibition of PI3K significantly sensitized skin cancer cells, and led to an enhanced CGE-induced apoptosis (Figure 5). These findings strongly suggest that CGE might serve as a chemo-preventive agent against skin cancer. The LC-MS/MS analysis showed that about 20% was chlorogenic acid and more than 70% of the partially purified CGE extract was maysin and its derivatives, which were the major active compounds, contains a mannose bound flavonoid backbone that may be easily absorbed in the body. Given this structural feature, maysin might contribute new insights into the treatment and/or preventive measures against cancer using natural compounds. Future studies should continue to investigate the pharmacological properties of CGE and assess its effectiveness as an anti-cancer agent.

## Abbreviations

CGE: Centipede grass extract; PI3K: Phosphoinositide 3-kinase; MTT: 3-(4, 5-dimethylthiazol-2-yl)-2, 5-di-phenyltetrazolium bromide; GSK3β: Glycogen synthase kinase-3 beta; PI: Propidium iodide; PARP: Poly-(ADP-ribose)-polymerase; PVDF: Polyvinyl difluoride; DiOC6 (3): 3, 30-dihexyloxacarboxyanine iodide; DMSO: Dimethyl sulphoxide; FITC: Fluorescein isothiocyanate.

## Competing interests

The authors declare that they have no competing interest.

## Authors’ contributions

BYC^1^ conceived this study and designed the experiments. SB and HWB performed most of the experiments and analysed data. CHP and DMJ performed FACS analysis and BYC helped to provide centipedegrass extracts. BYC^2^ and HWB supervised the project and wrote the manuscript with the help of SB, CHB, DMJ, and BYC^1^. All authors read and approved the final manuscript.

## Pre-publication history

The pre-publication history for this paper can be accessed here:

http://www.biomedcentral.com/1472-6882/13/350/prepub

## Supplementary Material

Additional file 1: Figure S1 TUNEL staining of SKMEL-5 skin cancer cells. Cells were treated with 50 μg·mL^-1^ CGE for 24 h and double staining with TUNEL and DAPI demonstrated an increase in the apoptotic cells population. Click here for file

Additional file 2: Figure S2 Effect of CGE on the apoptosis pathway. Cells were treated with 50 μg·mL^-1^ CGE for 24 h. Equal amounts of protein from each sample were separated by SDS-PAGE and immunoblotted. Click here for file

## References

[B1] Mantena SK Sharma SD Katiyar SK Berberine inhibits growth, induces G1 arrest and apoptosis in human epidermoid carcinoma A431 cells by regulating Cdki-Cdk-cyclin cascade, disruption of mitochondrial membrane potential and cleavage of caspase 3 and PARP Carcinogenesis 2006 13 2018 2027 10.1093/carcin/bgl043 16621886

[B2] Yusuf N Irby C Katiyar SK Elmets CA Photo protective effects of green tea polyphenols Photodermatol Photoimmunol Photomed 2007 13 48 56 10.1111/j.1600-0781.2007.00262.x 17254040

[B3] Hennessy BT Smith DL Ram PT Lu Y Mills GB Exploiting the PI3K/AKT pathway for cancer drug discovery Nat Rev Drug Discov 2005 13 988 1004 10.1038/nrd1902 16341064

[B4] Khaled M Larribere L Bille K Aberdam E Ortonne JP Ballotti R Bertolotto C Glycogen synthase kinase 3β is activated by cAMP and plays an active role in the regulation of melanogenesis J Biol Chem 2002 13 33690 33697 10.1074/jbc.M202939200 12093801

[B5] Barampuram S Chung BY Lee SS An BC Lee EM Cho JY Development of an embryogenic callus induction method for centipede grass (Eremochoa ophiuroides Munro) and subsequent plant regeneration In Vitro Cell Dev Biol Plant 2009 13 151 161

[B6] Johnson AW Influence of organic pesticides on nematode populations and seed production of centipede grass Journal of Nematolgy 1970 13 252 254 PMC2618751 19322306

[B7] Lee EM Lee SS Chung BY Cho JY Lee IC Ahn SR Jang SR Kim TH Pancreatic lipase inhibition by C-glycosidic flavones isolated from eremochloa ophiuroides Molecules 2010 13 8251 8259 10.3390/molecules15118251 21081855 PMC6259569

[B8] Park HJ Chung BY Lee MK Song Y Lee SS Chu GM Kang SN Song YM Kim GS Cho JH Centipede grass exerts anti-adipogenic activity through inhibition of C/EBPbeta, C/EBPalpha, and PPARgamma expression and the AKT signaling pathway in 3T3-L1 adipocytes BMC Complement Altern Med 2012 13 230 10.1186/1472-6882-12-230 23181522 PMC3584807

[B9] Mishra KP Padwad YS Dutta A Ganju L Sairam M Banerjee PK Sawhney RC Aqueous extract of Rhodiola imbricata rhizome inhibits proliferation of an erythroleukemic cell line K-562 by inducing apoptosis and cell cycle arrest at G2/M phase Immunobiology 2008 13 125 131 10.1016/j.imbio.2007.07.003 18241696

[B10] Oh SM Kim J Lee J Yi JM Oh DS Bang OS Kim NS Anticancer potential of an ethanol extract of Asiasari radix against HCT-116 human colon cancer cells in vitro Oncol Lett 2013 13 305 310 23255939 10.3892/ol.2012.1012 PMC3525467

[B11] Pan TL Wang PW Leu YL Wu TH Inhibitory effects of Scutellaria baicalensis extract on hepatic stellate cells through inducing G2/M cell cycle arrest and activating ERK-dependent apoptosis via Bax and caspase pathway J Ethnopharmacol 2012 13 829 837 10.1016/j.jep.2011.12.028 22210104

[B12] Sunderraja S Thangama R Vellingiri S Krishnaswamy K Palani G Shanmugam A Soundarapandian K **γ**-**Sitosterol from Acacia nilotica L. induces G2**/**M cell cycle arrest and apoptosis through c**-**Myc suppression in MCF**-**7 and A549 cells** J Ethnopharmacol 2012 13 803 809 10.1016/j.jep.2012.03.014 22440953

[B13] Brunelle JK Letai A Control of mitochondrial apoptosis by the Bcl-2 family J Cell Sci 2009 13 437 441 10.1242/jcs.031682 19193868 PMC2714431

[B14] Zhong ZG Wu DP Huang JL Liang H Pan ZH Zhang WY Lu HM Progallin A isolated from the acetic ether part of the leaves of Phyllanthus emblica L. induces apoptosis of human hepatocellular carcinoma BEL-7404 cells by up-regulation of Bax expression and down regulation of Bcl-2 expression J Ethnopharmacol 2011 13 765 772 10.1016/j.jep.2010.11.001 21073944

[B15] Chang HY Yang X Proteases for cell suicide functions and regulation of caspases Microbiol Mol Biol Rev 2000 13 821 846 10.1128/MMBR.64.4.821-846.2000 11104820 PMC99015

[B16] Oliver L Vallette FM The role of caspases in cell death and differentiation Drug Resist Updat 2005 13 163 170 10.1016/j.drup.2005.05.001 15946892

[B17] Chen X Wang J Qin Q Jiang Y Yang G Rao K Wang Q Xiong W Yuan J Mono-2-ethylhexyl phthalate induced loss of mitochondrial membrane potential and activation of Caspase3 in HepG2 cells Environ Toxicol Pharmacol 2012 13 421 430 10.1016/j.etap.2012.02.001 22387354

[B18] Wills PJ Asha VV Chemo preventive action of Lygodiumflexuosum extract in human hepatoma PLC/PRF/5 and Hep 3B cells J Ethnopharmacol 2009 13 294 303 10.1016/j.jep.2009.01.006 19168119

[B19] Kirs N Patrizia A Bcl-2 family members: essential players in skin cancer Cancer Lett 2012 13 1 13 10.1016/j.canlet.2012.01.031 22281242

[B20] Choi MJ Park EJ Oh JH Min KJ Yang ES Kim YH Lee TJ Kim SH Choi YH Park JW Kwon TK Cafestol, a coffee-specific diterpene, induces apoptosis in renal carcinoma Caki cells through down-regulation of anti-apoptotic proteins and Akt phosphorylation Chem Biol Interact 2011 13 102 108 10.1016/j.cbi.2011.02.013 21334318

[B21] Kim HG Song H Yoon DH Song BW Park SM Sung GH Cordyceps pruinosa extracts induce apoptosis of HeLa cells by a caspase dependent pathway J Ethnopharmacol 2010 13 342 351 10.1016/j.jep.2010.01.049 20138133

[B22] Oh JH Lee TJ Kim SH Choi YH Lee SH Lee JM Kim YH Park JW Kwon TK Apoptosis induction of apoptosis by withaferin a in human leukemia U937 cells through down regulation of Akt phosphorylation Apoptosis 2008 13 1494 1504 10.1007/s10495-008-0273-y 19002588

[B23] Arafael-SA ZQ Barakat BM Wani G Zhao Q Ei-Mahdy MA Wani AA Tangeretin sensitizes cisplatin-resistant human ovarian cancer cells through down regulation of phosphoinositide 3-kinase/Akt signaling pathway Cancer Res 2009 13 8910 8917 10.1158/0008-5472.CAN-09-1543 19903849 PMC3319094

[B24] Desagher S Martinou JC Mitochondria as the central control point of apoptosis Trends Cell Biol 2000 13 369 377 10.1016/S0962-8924(00)01803-1 10932094

[B25] Kohler C Orrenius S Zhivotovsky B Evaluation of caspase activity in apoptotic cells J Immunol Methods 2002 13 97 110 10.1016/S0022-1759(02)00073-X 12072181

[B26] Mohapatra S Chu B Zhao X Djeu J Cheng JQ Pledger WJ Apoptosis of metastatic prostate cancer cells by a combination of cyclin-dependent kinase and AKT inhibitors Int J Biochem Cell Biol 2009 13 595 602 10.1016/j.biocel.2008.07.013 18708158

[B27] Chang CY Shen CC Su HL Chen CJ Gefitinib induces apoptosis in human glioma cells by targeting bad phosphorylation J Neurooncol 2011 13 507 522 10.1007/s11060-011-0632-3 21744078

[B28] Dharmalingam NG Perma E Ramachandaran A Kalimuthula S Jagadeesan A Induction of apoptosis and inhibition of PI3K/Akt pathway in PC-3 and LNCaP prostate cancer cells by ethanolic neem leaf extract J Ethnopharmacol 2011 13 644 650 10.1016/j.jep.2011.01.015 21277364

[B29] Sakamaki J Daitoku H Ueno K Hagiwara A Yamagata K Fukamizu A Arginine methylation of BCL-2 antagonist of cell death (BAD) counteracts its phosphorylation and inactivation by Akt Proc Natl Acad Sci U S A 2011 13 6085 6090 10.1073/pnas.1015328108 21444773 PMC3076815

[B30] Grimes CA Jope RS The multi-faceted roles of glycogen synthase kinase-3beta in cellular signaling Prog Neurobiol 2001 13 391 426 10.1016/S0301-0082(01)00011-9 11527574

[B31] Havasi A Li Z Wang Z Martin JL Botla V Ruchalski K Schwartz JH Borkan SC Hsp27 inhibits Bax activation and apoptosis via a phosphatidylinositol 3-kinase-dependent mechanism J Biol Chem 2008 13 2305 2313 10.1074/jbc.M801291200 PMC2431006 18299320

[B32] Lama D Sankararamakrishnan R Anti-apoptotic Bcl-XL protein in complex with BH3peptides of pro-apoptotic Bak, Bad, and Bim proteins: comparative molecular dynamics simulations Proteins 2008 13 492 514 10.1002/prot.22075 18452209

