# Supplementary figures and images for: Molecular mechanism of apoptosis induction in skin cancer cells by the centipedegrass extract
Source: BMC Complement Altern Med. 2013 Dec 11;13:350. doi: 10.1186/1472-6882-13-350 (PMC3880216; doi:10.1186/1472-6882-13-350)

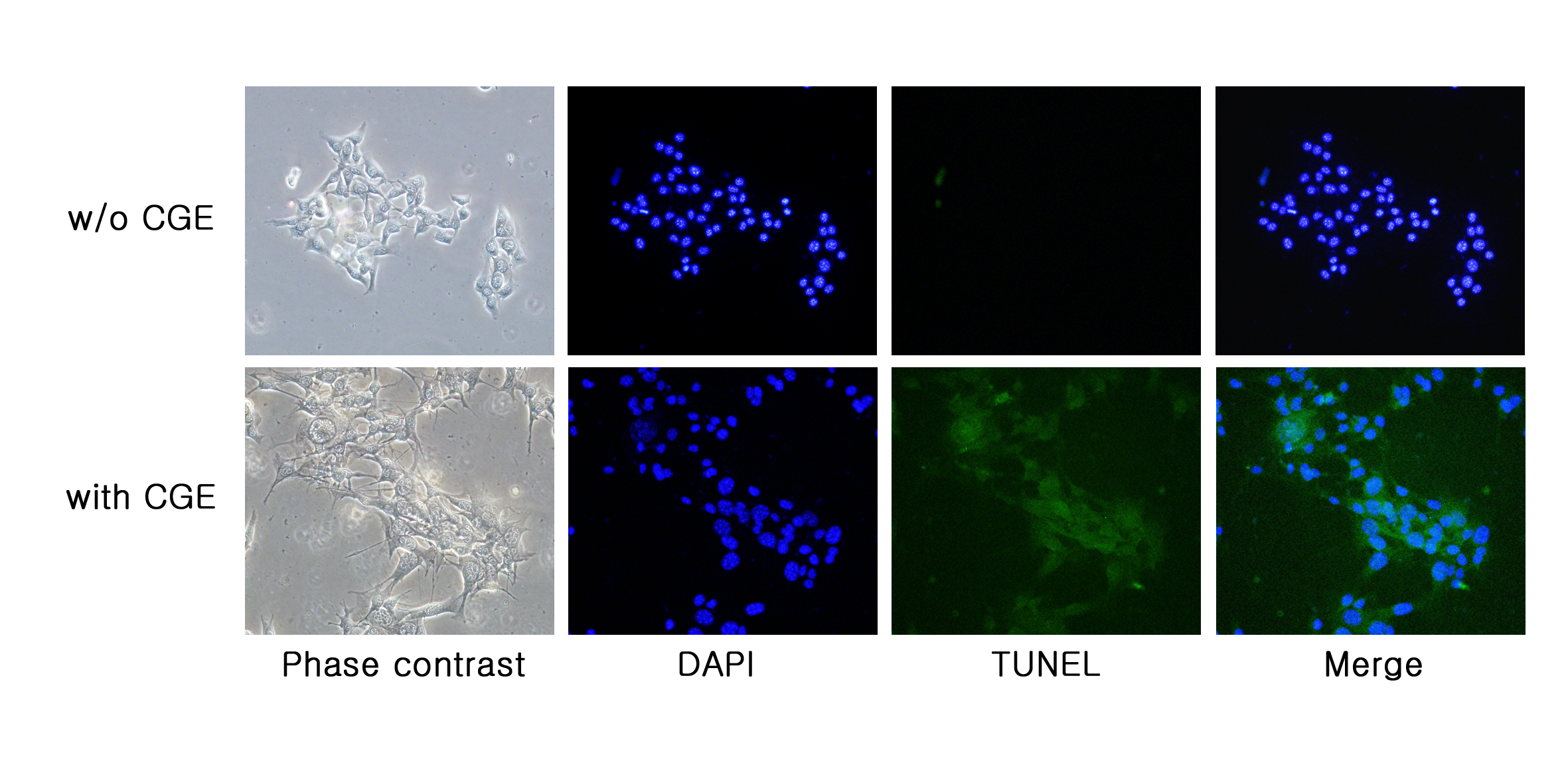

Supplement: Additional file 1: Figure S1 — TUNEL staining of SKMEL-5 skin cancer cells. Cells were treated with 50 μg·mL-1 CGE for 24 h and double staining with TUNEL and DAPI demonstrated an increase in the apoptotic cells population. [file 1472-6882-13-350-S1.tiff]

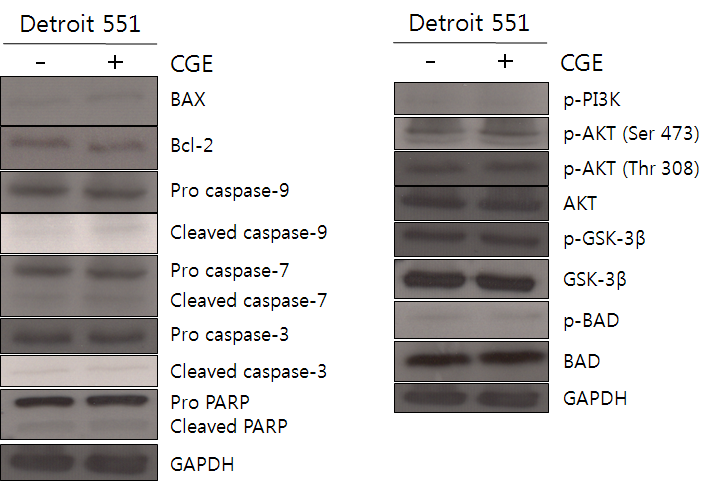

Supplement: Additional file 2: Figure S2 — Effect of CGE on the apoptosis pathway. Cells were treated with 50 μg·mL-1 CGE for 24 h. Equal amounts of protein from each sample were separated by SDS-PAGE and immunoblotted. [file 1472-6882-13-350-S2.tiff]
